# Patient Outcomes in Association With Significant Other Responses to Chronic Fatigue Syndrome: A Systematic Review of the Literature

**DOI:** 10.1111/cpsp.12093

**Published:** 2015-03-14

**Authors:** Rebecca Band, Alison Wearden, Christine Barrowclough

**Affiliations:** School of Psychological Sciences & Manchester Centre for Health Psychology, University of Manchester

**Keywords:** behaviors, beliefs, chronic fatigue syndrome, significant others

## Abstract

Social processes have been suggested as important in the maintenance of chronic fatigue syndrome (also known as myalgic encephalomyelitis; CFS/ME), but the specific role of close interpersonal relationships remains unclear. We reviewed 14 articles investigating significant other responses to close others with CFS/ME and the relationships between these responses and patient outcomes. Significant other beliefs attributing patient responsibility for the onset and ongoing symptoms of CFS/ME were associated with increased patient distress. Increased symptom severity, disability, and distress were also associated with both solicitous and negative significant other responses. Specific aspects of dyadic relationship quality, including high Expressed Emotion, were identified as important. We propose extending current theoretical models of CFS/ME to include two potential perpetuating interpersonal processes; the evidence reviewed suggests that the development of significant other–focused interventions may also be beneficial.

Patients diagnosed with chronic fatigue syndrome (also known as myalgic encephalomyelitis; CFS/ME) experience severe fatigue not attributable to alternative medical and psychiatric diagnoses (Fukuda et al., 1994). CFS/ME is associated with high levels of patient disability and healthcare use (McCrone, Darbishire, Ridsdale, & Seed, 2003). The worldwide prevalence is currently estimated to vary from 0.2% to 2.6% within adult populations (Prins, van der Meer, & Bleijenberg, 2006). This article seeks to identify and review empirical studies that have examined significant other responses to CFS/ME and the associations of these responses with patient outcomes.

Cognitive-behavioral models provide an explanatory framework for the development and maintenance of CFS/ME and distinguish between the predisposing, precipitating, and perpetuating factors (Surawy, Hackmann, Hawton, & Sharpe, 1995). These models propose that failure to recover from an initial illness trigger gives rise over time to a state of persistent physiological dysregulation, involving muscular and cardiovascular deconditioning and disturbed sleep (Deary, Chalder, & Sharpe, 2007). This dysregulation is maintained by the action of a complex set of interacting variables, which may include physiological, cognitive, behavioral, emotional, and social factors (Deary et al., 2007; Surawy et al., 1995).

The importance of cognitive processes in the development and maintenance of CFS/ME and their association with patient behavioral responses to symptom experience has been described (Moss-Morris, 2005). As the illness develops, long periods of rest may be interspersed with short bursts of exertion, referred to as all-or-nothing behavior (Moss-Morris, Spence, & Hou, 2011; Spence, Moss-Morris, & Chalder, 2005). These behaviors exacerbate fatigue severity and increase symptom focusing (Moss-Morris, 2005). Patients who view symptoms as indicative of ongoing pathological damage in the body engage in higher levels of activity limitation—termed *fear avoidance*—as a strategy to prevent further fatigue (Deale, Chalder, & Wessely, 1998). This in turn perpetuates deconditioning and further reduces tolerance for activity (Schmaling, Fiedelak, Bader, & Buchwald, 2005). Symptom focusing and negative beliefs about the condition have been associated with poorer patient outcomes (Moss-Morris & Chalder, 2003; Moss-Morris, Sharon, Tobin, & Baldi, 2005). Cognitive-behavioral therapy (CBT) and graded exercise therapy (GET) are effective in reducing fatigue and improving patient functioning (Castell, Kazantzis, & Moss-Morris, 2011). Changes in beliefs about activity and symptom preoccupation have been identified as key mechanisms for improvement (Moss-Morris et al., 2005; Wearden & Emsley, 2013; Wiborg, Knoop, Stulemeijer, Prins, & Bleijenberg, 2010).

Patients with CFS/ME experience high levels of stigma (Looper & Kirmayer, 2004) and a reduction in opportunity for wider social contact (Assefi, Coy, Uslan, Smith, & Buchwald, 2003), which may amplify any effect of interpersonal variables in close relationships with significant others. Patients report that they are distressed when their significant others do not understand their illness or validate their suffering (Dickson, Knussen, & Flowers, 2007). Just as patients’ illness beliefs are thought to generate behavioral and emotional responses to illness events and to guide coping strategies (Leventhal, Meyer, & Nerenz, 1980), significant others’ beliefs about patients’ illnesses are thought to affect their emotional responses (Dempster et al., 2011). CFS/ME patients and their significant others may establish a joint narrative or understanding of the condition (Brooks, King, & Wearden, 2013) or alternatively may experience some level of discord about the meaning of symptoms and how to manage them (Dickson et al., 2007). There is growing evidence from studies in various health conditions that dyadic belief congruence may have important associations with various adaptive outcomes, including patient and significant other distress, and perceived support (Cano, Johansen, & Geisser, 2004; Figueiras & Weinman, 2003; Martire et al., 2006; Sterba et al., 2008). It has therefore been suggested that social factors such as the effect of others’ beliefs about the legitimacy of the illness could be incorporated into cognitive-behavioral models of CFS/ME in order to both aid understanding of symptom maintenance and inform potential interpersonal interventions (Moss-Morris, Deary, & Castell, 2013). In sum, examining significant other factors, particularly significant other beliefs and responses to patients’ symptoms and illness, in association with patient outcomes may be beneficial to further advancing our understanding of the perpetuating role of interpersonal factors in CFS/ME.

## Aims

The review will address two main aims, first relating to significant other responses to the condition, and second, the associations of significant other beliefs, behavioral responses, and dyadic relationship satisfaction to patient outcomes such as symptom severity, physical functioning, and psychological adaptation.

## Method

### Search Procedure

The following electronic databases were searched: PsycINFO, MEDLINE, EMBASE, CINAHL, Web of Knowledge/Science, PubMed, and the Cochrane Library. Further articles and unpublished manuscripts were sought by examining the reference lists of identified articles, in addition to seeking consultation with experts in the field. The search was completed in December 2012 and updated in March 2014 and followed a two-stage procedure; first, specific CFS/ME and significant other population terms were searched (see Table 1). Subsequently, these were combined with significant other response variable terms.

**Table 1:** Review search terms, inclusion criteria, and patient outcomes relevant to article selection

CFS/ME population terms	
Chronic fatigue syndrome/ CFS/ Myalgic encephalomyelitis/ ME/ Chronic fatigue and immune dysfunction syndrome/ CFIDS/ Post viral fatigue syndrome	
Significant other population terms	
Significant other/ carer/caregiver/ partner/ spouse/ wife/ husband/ family member/ parent/ mother/ father/ daughter/ son/ child	
Significant other response variable terms	
Illness representation/ cognitive representation/ common-sense model/ illness perception/ attribution/ solicitous/ distracting/ punishing/ facilitating/ belief/ emotion/ expressed emotion/ EE/ criticism/ critical comments/ hostility/ warmth/ over-involvement/ overprotection	
Inclusion criteria	
Adults (aged 16+) who had received a specialist clinician diagnosis of CFS/ME	
Assess significant other beliefs or responses to CFS/ME	
Assess significant other variables in association with patient outcomes	
Articles published in English	
Any significant other relationship	
Patient outcomes (with examples)	
Symptom severity	Fatigue, pain, other CFS symptoms
Physical functioning	Disability, physical activity, rest, functional abilities, daily activities
Psychological adjustment	Depression, anxiety, distress, adjustment to illness
Relationship satisfaction	Happiness, satisfaction, adjustment
Significant other predictor variables (with examples)	
Illness beliefs	Causal attributions, illness perceptions
Behavioral responses	Solicitous, distracting, punishing, facilitating
Affect	Anxiety, depression, distress, anger, irritation
Expressed Emotion	Emotional over-involvement, criticism, hostility, warmth

### Article Selection

To be eligible, articles had to include patients who had received a physician diagnosis of CFS/ME, chronic fatigue and immune dysfunction syndrome, post-viral fatigue syndrome, or idiopathic chronic fatigue; use of the Oxford (Sharpe et al., 1991) or Center for Disease Control (Fukuda et al., 1994) criteria for CFS/ME was noted.

Qualitative and quantitative studies addressing one of the two review aims were considered; broad inclusion criteria were selected to maximize potential article inclusion. No exclusion criterion was set with reference to the nature of the significant other relationship. Studies were excluded if they contained mixed samples where the CFS/ME sample was indistinguishable from other participant groups, and if participants were entirely or predominantly children (aged 16 or under). Only articles published following the first modern definition of CFS (Holmes et al., 1988) were sought and included.

**Figure 1:**
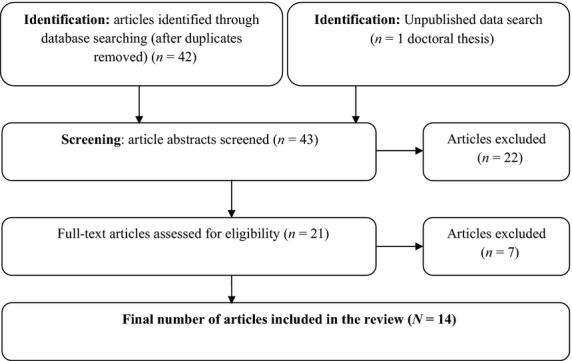
Flowchart illustrating the stages of article selection and data extraction for the review.

Figure1 demonstrates the number of studies included and excluded at each stage of the identification and screening process. One abstract identified referred to work in progress; a full-text version could not be obtained. Articles that did not meet the review objectives were excluded, in addition to articles that met exclusionary criteria. Five articles were identified that assessed significant other responses to CFS/ME. Nine articles were identified that assessed patient outcomes in association with significant other predictor variables. To assess reliability, a second doctoral psychology student also selected articles to be included in the final synthesis. In the event of disagreement, discussion between raters was used to reach consensus. A quality assessment was conducted using a tool specifically designed for use with contributing articles with diverse methodological designs (Sirriyeh, Lawton, Gardner, & Armitage, 2012). The measure has demonstrated adequate reliability (Sirriyeh et al., 2012), although normative values associated with study quality are not currently available. Second quality assessments were also conducted to avoid rating bias.

### Data Extraction and Synthesis

Table 2 provides an overview of the relevant data extracted from each included article, identifying both the significant other response variables and patient outcomes reported within each study, where applicable, and providing the overall quality rating. The major findings emerging from the data are synthesized below.

**Table 2:** Summary of studies examining significant other responses to CFS/ME and the association with patient outcomes

	Demographics								Study Assessment			
	Patient			Significant Other			SO Type (%)					
Study	*N* and CFS Definition	% Female	Age (Mean)	*N*	% Male	Age (Mean)	Partner	Parent	SO Variables (Measure)	Patient Outcomes (Measure)	Key Results	Study Quality (%)
Ax (1999)	155 with medical professional diagnosis of CFS/ME/PVF	76.8	40.4	94	50.5	48.45	62	32	Coping strategies^1^ (WCQ)	–	SO coping strategies influenced by gender; females reported higher levels of problem- and emotion-focused coping strategies, and relationship type (parent or partner) was also important	71
Ax et al. (2002)	n/a			17	71	44	71	6	Coping^2^ Illness adjustment^2^	–	Difficulties are highlighted, but become more manageable over time with increased adjustment	69
Band et al. (2014)	55 Oxford criteria	91	38	55	49	48	55	36	Expressed Emotion (Criticism and emotional over-involvement)^2^ (CFI)	Fatigue severity^1^ (CF, VAS) Disability (physical functioning)^1^ (SF-36) Depression^1^ (HADS)	High SO criticism and emotional over-involvement predictive of greater fatigue severity longitudinally. Increased patient depression mediated the association between criticism and fatigue severity	74
Blazquez et al. (2012)	40 CDC criteria	100	41	40	100	44.6	100		Relationship adjustment^1^ (32-item DAS)	Cardio-respiratory responses³	Dyadic relationship adjustment variables were associated with patient indicators of functional capacity (i.e., cardio-respiratory responses) at resting and low-intensity activity levels	50
Brooks, Daglish, et al. (2013), Brooks, King, et al. (2013)	30 CDC criteria	73.3	41	30	40	48	60	33.3	Attributional beliefs² (LACS) Response styles^1^ (FRQ) Distress^1^ (GHQ) Relationship satisfaction^1^ (6-point VAS)	Fatigue severity^1^ (CF) Disability (physical functioning)^1^ (SF-36)	Increased SO distress and negative responses associated with attributing illness events to personal and internal patient factors. Encouragement to rest responses associated with SO controllability attributions and poorer patient outcomes	65
Butler et al. (1999)	50 Oxford criteria	56	39	44	50	39.6	100		Attributional beliefs^1^ (modified SIQ)	–	SOs made predominantly normalizing attributions for their own symptoms but somatic attributions for patient symptoms, in line with patient beliefs	67
Goodwin (1997)	131 with medical professional diagnosis of CFS	100	42.9	131	100	45	100		Marital adjustment: Empathy^1^ (DPT) Support and conflict (IPRI)	Number of symptoms^1^ Problem symptoms^1^ (DCFSSS)	Increased problem symptoms associated with reduced marital adjustment and high conflict. Higher number of symptoms associated with lower SO empathy	48
Goodwin (2000)	131 with medical professional diagnosis of CFS	100	42.9	131	100	45	100		Marital adjustment: Empathy^1^ (DPT) Support and conflict (IPRI)	Symptom transition^1^ (DPSTS)	Increased patient symptom transition was associated with high conflict, reduced marital adjustment, and reduced SO support and empathy	45
Heijmans et al. (1999)	49 with medical professional diagnosis of CFS	92	40.4	49	–	42.7	100		Illness representations^1^ (IPQ)	Disability^1^ (SF-36)	Dyadic differences in timeline beliefs; shorter SO timeline beliefs associated with better patient functioning. Poorer outcomes associated with reduced SO biological causal beliefs	52
Kelly et al. (1999)	41 (diagnostic criteria not reported)	83	46	25	–	–			Illness beliefs^1^ Impact of CFS/ME^1^ Social support^1^ (ISSB)	Depression^1^ (BDI, POMS) Stress^1^ (PSS)	High prevalence of negative consequences for SOs. No difference in level of support reported according to SO causal beliefs. No differences in patient outcomes for those with/without support	48
Richards et al. (2006)	21 Oxford criteria	63	17^a^	21	–	–		100	Illness beliefs²	–	Highly similar patient and SO beliefs about onset; preference for physical causes with some psychosocial causes identified	55
Romano et al. (2009)	111 CDC 6 met criteria for idiopathic CF	93	44.4	94	63	46.2	75	3	Response styles (patient reported)^1^ (MPI) Relationship satisfaction^1^ (7-item DAS) Perceived illness behaviors^1^ (PBC) Observed responses^4^	Fatigue severity^1^ (1-item MAF) Disability (physical functioning)^1^ (SF-36) Pain intensity^1^ (11-point VAS) Depression^1^ (CES-D) Observed illness behaviors^4^	Solicitous responses associated with poorer physical functioning. Negative responses associated with increased patient depression. Observational data did not replicate self-reported associations; observed negative responses were associated with decreased patient illness behaviors and better physical functioning	67
Schmaling et al. (2000)	119 CDC criteria	76	39.0		n/a		100		Response styles (patient reported)^1^ (MPI)	Fatigue severity^1^ (16-item MAF) Disability (physical functioning)^1^ (SF-36)	Solicitous responses associated with greater fatigue and bodily pain; moderated by relationship satisfaction. High relationship satisfaction associated with increased disability	62
White et al. (2006)	105 with medical professional diagnosis and CDC criteria	88	47	87	–	50	60		Attributional beliefs^1^ Response styles (SO reported)^1^ (SSBQ)	Psychological adjustment^1^ (BSI)	SO causal beliefs for internal patient factors associated with poorer psychological outcomes and more unhelpful SO responses. Unhelpful SO responses associated with increased anxiety and depression	53

*Note*. Dashes indicate where information was not reported. SO denotes significant other. SO variable and patient outcome assessment: ^1^indicates measured by questionnaire measure; ^2^indicates measured by interview; ^3^indicates measured by patient physical task; ^4^indicates measured by dyadic observational task. BDI = Beck Depression Inventory; BSI = Brief Symptom Inventory; CES-D = Center for Epidemiological Studies Depression scale; CF = Chalder Fatigue scale; CFI = Camberwell Family Interview; DAS = Dyadic Adjustment Scale; DCFSSS = De Groot Chronic Fatigue Syndrome Symptom Scale; DPSTS = De Groot Perceived Symptom Transition Scale; DPT = Dyadic Perspective Taking questionnaire; FRQ = Family Response Questionnaire; GHQ = General Health Questionnaire; HADS = Hospital and Anxiety Questionnaire; IPQ = Illness Perception Questionnaire; IPRI = Interpersonal Relationship Inventory; ISSB = Inventory of Socially Supportive Behaviours; LACS = Leeds Attributional Coding System; MAF = Multidimensional Assessment of Fatigue; MPI = Multidimensional Pain Inventory; PBC = Pain Behaviour Checklist; POMS = Profile of Mood States; PSS = Perceived Stress Scale; SF-36 = Short Form (36) Health Survey; SIQ = Symptom Interpretation Questionnaire; SSBQ = Social Support Behaviour Questionnaire; VAS = Visual Analogue Scale; WCQ = Ways of Coping Questionnaire.

a Median value.

## Results

### Significant Other Responses to CFS/ME

Significant others experience negative consequences as a result of the condition; over half report CFS/ME has had a negative impact upon their life and their relationship with the patient (Kelly, Soderlund, Albert, & McGarrahan, 1999). Negative emotional outcomes and relationship difficulties have been reported, such as experiencing guilt or embarrassment (Ax, Gregg, & Jones, 2002). Further practical limitations and obstacles within family life are raised, for example, disruption of family roles, financial difficulties, and anger experienced as a result of the condition (Ax et al., 2002; Kelly et al., 1999). However, significant others retrospectively report that they manage these difficulties more effectively over time. Initial optimism that the condition would improve gives way to acceptance, although doubts over the legitimacy of the condition are occasionally raised across the illness course (Ax et al., 2002).

Significant other coping appears to be influenced by gender and the nature of the relationship with the patient. Overall, female significant others engage in higher levels of both distress reduction (emotion-focused) and stress reduction (problem-focused) coping strategies than their male counterparts (Ax, 1999). Problem-focused coping may include cognitive or behavioral strategies that attempt to alter the source of the stress in the environment, such as making a plan to deal with the illness, while emotion-focused coping includes attempts at regulating the emotional distress associated with the stressor, such as trying to forget about the situation (Folkman & Lazarus, 1980). The patient–significant other relationship type also impacted upon significant other coping strategies; parents report using more stress reduction coping strategies than spouses. Furthermore, significant gender differences in distress reduction strategies were revealed within married dyads. Husbands reported reduced levels of distress reduction techniques, resulting in greater disparity in coping strategies within the dyad compared to those partner dyads where the significant other was female (Ax, 1999).

### Significant Other Beliefs

#### Significant Other Causal Attributions for CFS/ME

Patients and significant others consistently report a predominant preference for physical factors in explaining the illness onset, with factors such as infection or disordered immune systems being reported most often (Richards, Chaplin, Starkey, & Turk, 2006). Many significant others also select a combination of causal factors (Kelly et al., 1999). Stress, including family-related stressors, was also often implicated in significant other psychological explanations (Richards et al., 2006). However, significant others were identified to endorse all causal factors (i.e., viral, external, and internal) less strongly, relative to the patient (White, Lehman, Hemphill, Mandel, & Lehman, 2006). These findings were observed in parents (Richards et al., 2006) and in a predominantly partner sample (White et al., 2006).

Significant others who attributed the onset of the condition to internal patient factors showed increased levels of unhelpful responses (White et al., 2006), specifically behaviors such as encouraging patients to overcome the situation or acting in a forced cheerful way (Johnson, Hobfoll, & Zalcberg-Linetzy, 1993). However, no differences were found in the level of social support provided by significant others when comparing individuals with physical to physical–psychological casual explanations for CFS/ME (Kelly et al., 1999). Methodological differences in the measurement of beliefs and responses may be, in part, responsible for the apparent inconsistencies. The way beliefs were classified and the number of items used to assess illness beliefs varied between studies (Kelly et al., 1999; White et al., 2006); for example, physical versus psychological causes were rated on four items (Kelly et al., 1999) compared to internal versus external causal factors, rated by 11 items (White et al., 2006). Direct comparison of these scales is not possible, as the Kelly et al. (1999) scale items are not reported.

#### Attributions for Patient Symptoms and Illness Events

Significant others also demonstrate highly similar illness beliefs to those held by the patient (Heijmans, de Ridder, & Bensing, 1999), with further congruence identified in relation to patient symptom experience (Butler, Chalder, & Wessely, 2001). Butler et al. (2001) asked both patients and significant others to report the likely cause for their own and their significant others’ common physical symptom experience. Potential attributions can be ascribed to either physical (somatic) causes, which reflect beliefs that there is something wrong within the body, psychological causes, or, most commonly, environmental (normalized) attributions (Robbins & Kirmayer, 1991). CFS/ME patients tended to make somatic attributions for all symptoms, including those they had never experienced. Significant others demonstrated a typical normalizing attributional style for their own symptom experience, yet attributed patient symptoms to physical abnormalities most often, in line with patient beliefs (Butler et al., 2001). These studies would seem to confirm high concordance of dyadic beliefs about patient symptoms and CFS/ME illness events, but are applicable in the context of patient–partner dyads only.

Furthermore, significant other attributions for symptom events have been linked with significant other emotions and behavioral responses (Brooks, Daglish, & Wearden, 2013). Significant others who attributed negative symptom changes and illness events to personal and internal patient factors were more distressed (Brooks, Daglish, et al., 2013). These attributions were also associated with significant other rejecting-hostile responses on the Family Response Questionnaire (FRQ; Cordingley, Wearden, Appleby, & Fisher, 2001). Additionally, beliefs that attributed high levels of patient controllability over symptoms and illness events were associated with higher levels of significant other encouragement to rest (Brooks, Daglish, et al., 2013).

Further methodological considerations regarding the measurement of significant other responses may also explain inconsistencies across the literature. Significant associations between significant other beliefs and responses were only identified when examining specific significant other behavioral response styles (Brooks, Daglish, et al., 2013; White et al., 2006) rather than overall level of social support (Kelly et al., 1999). Precise measurement of beliefs, behaviors, and outcome variables may lead to a better understanding of the correlates of these interpersonal processes in CFS/ME and enable direct comparisons between studies.

#### Significant Other Beliefs and Patient Outcomes

The nature and content of significant other beliefs have been considered, yet the extent to which these impact upon long-term patient outcomes remains relatively under researched. Only two articles published to date have examined the association between significant other beliefs and patient outcomes (Heijmans et al., 1999; White et al., 2006). We know that both patients and significant others tend to make physical causal attributions for the onset of the condition (Richards et al., 2006; White et al., 2006). However, increased levels of patient anxiety, depression, and rumination were associated with significant others holding stronger beliefs that the CFS/ME onset was due to internal patient factors, such as stress or overwork (White et al., 2006). Attributions made to external or viral factors did not relate to patient outcomes (White et al., 2006). Similarly, significant other beliefs that minimized physical causes for the illness onset were also associated with poorer patient social functioning and vitality (Heijmans et al., 1999). It is possible that significant others identify poorer psychosocial functioning in patients and therefore attribute the illness to these factors. The evidence suggests that attributing responsibility to the patient for any aspect of the condition is associated with increased distress for both patients and significant others (Brooks, Daglish, et al., 2013; White et al., 2006); this association appears to be applicable to both parent and partner significant other subgroups.

Alternatively, significant other endorsement of internal or psychological causal factors would likely be discordant with patient models of their illness (i.e., arising as a result of physical or external factors), which may account for poorer patient outcomes. However, Heijmans et al. (1999) examined dyadic illness representations using the Illness Perception Questionnaire (IPQ; Weinman, Petrie, Moss-Morris, & Horne, 1996) and identified that significant other beliefs in a shorter illness timeline, relative to the patient, were actually correlated with better patient functioning (activity, psychological adjustment, and vitality). Further investigation of the impact of dyadic belief congruence or incongruence in CFS/ME is warranted.

### Significant Other Behavioral Responses

#### Reinforcing (or Solicitous) Significant Other Responses

Patients who perceived high levels of significant other solicitous behavior also reported higher levels of fatigue severity and bodily pain (Schmaling, Smith, & Buchwald, 2000), as well as worse levels of disability (Romano, Jensen, Schmaling, Hops, & Buchwald, 2009). These responses may include behaviors such as assisting, or doing tasks for the patient (Kerns, Turk, & Rudy, 1985). However, Romano et al. (2009) found no significant associations between solicitous responses and patient-reported fatigue severity. In both of these studies, significant other responses are measured with respect to how likely the significant other is to make the response in general, while a single item was used to assess level of fatigue severity at the time of responding (Romano et al., 2009), potentially accounting for inconsistencies between studies. Alternatively, these findings may reflect the demographics of the significant other groups included within these studies, comparing associations across a mixed significant other sample (i.e., not limited to partner relationships; Romano et al., 2009) and a partner-only sample (Schmaling et al., 2000). It is likely that relationship type may be important when considering the associations between significant other behavioral responses and patient outcomes.

Solicitous responses were also found to predict patient-reported illness behaviors, such as seeking help or expressions of fatigue and pain (Romano et al., 2009). Further evidence using an alternative classification of significant other behavioral responses has been obtained recently by Brooks, Daglish, et al. (2013). Increased patient disability was also found to be associated with high levels of significant other “active engagement,” a response style that includes behaviors such as finding out about and discussing the illness with the patient (Cordingley et al., 2001). Finally, significantly increased levels of patient fatigue and disability across the sample were associated with significant others encouraging patients to engage in rest (Brooks, Daglish, et al., 2013).

#### Negative Significant Other Responses

Negative responses (sometimes called punishing responses) include behaviors such as expressing irritation, frustration, or anger toward the patient or leaving the room (Kerns et al., 1985). Elevated levels of patient depression were associated with perceptions of negative responses from their significant others (Romano et al., 2009). Negative responses were also significant predictors of both increased patient depression and patient illness behaviors (Romano et al., 2009). Additionally, using an alternative classification of significant other responses, patient-perceived “unhelpful” responses were also associated with elevated patient anxiety and depression (White et al., 2006). The constituent behaviors for this subscale (Social Support Behaviour Questionnaire [SSBQ]; Johnson et al., 1993), such as trying to be cheerful despite the situation or offering the patient advice, seem to reflect a set of normalizing responses. These responses are different from the negative response classification as assessed via the Multidimensional Pain Inventory (MPI; Kerns et al., 1985) subscale, yet these “unhelpful” responses also provoke increased psychological distress within this population. These “unhelpful” responses are therefore potentially being interpreted in a similar negative manner, possibly as demonstrating a lack of empathy or invalidation of the patient's suffering by the significant other. However, the self-report trends were not replicated when observing dyadic interactions; observed negative responses were actually associated with reduced patient-reported disability (Romano et al., 2009).

### Relationship Quality

#### Low Relationship Quality

Increased symptom levels (total number of symptoms experienced and level of problematic symptoms) were found to be associated with lower overall relationship adjustment, as reported by the patient, by the significant other, and within the dyad (Goodwin, 1997). Similarly, increased symptom transition (i.e., pattern, frequency, duration of symptoms) was also associated with lower relationship adjustment (Goodwin, 2000). Goodwin (1997) identified that reports of marital adjustment were comparable for both patient and significant others and, additionally, that patient outcomes were predicted by both patient and significant other relationship variables. However, limitations with these studies must be noted; these studies were rated as being some of the poorest quality of those included within the synthesis. In addition, both measures of “problem symptoms” and “symptom transition” are taken from unpublished doctoral data, and therefore, no previous psychometric data on these measures exist; the results must be considered in line with these limitations.

In addition to self-reported patient outcomes, a recent study demonstrated an empirical association between relationship quality and observable measures of patient functional capacity (Blazquez, Guillamo, Alegre, Ruiz, & Javierre, 2012). A number of patient cardio-respiratory responses, such as heart rate and oxygen intake during breathing, taken at rest and low activity, were examined in association with both patient- and significant other–reported relationship adjustment. Poorer dyadic adjustment was associated with poorer ventilatory efficiency at rest, although this association during activity was only observed for those patients who were high in anxiety (Blazquez et al., 2012); it is conceivable that patients’ anxiety may also be highly correlated with relationship satisfaction. The findings would seem to suggest that the patient–significant other relationship may be interacting with psychobiological factors to impact upon health outcomes within this group, although further evidence would be necessary to establish these relationships more clearly and the implications for patient illness outcomes.

The studies examining relationship quality have done so only in the context of heterosexual, partnered dyads where the patient is female (Blazquez et al., 2012; Goodwin, 1997, 2000), limiting the applicability of these findings to this specific subgroup only. However, it has been noted elsewhere that significant others who report low levels of relationship happiness also experience high levels of distress and increased reporting of “concern for self” responses (Brooks, Daglish, et al., 2013). The association between low significant other relationship happiness and specific behavioral responses offers a potential explanation for the association between low relationship quality and increased symptom severity. These results suggest that relationship quality is important for both patient and significant other well-being (Brooks, Daglish, et al., 2013).

#### High Relationship Quality

High levels of patient-reported relationship satisfaction have been found to be associated with high levels of patient disability (Schmaling et al., 2000). Furthermore, relationship satisfaction was found to moderate the association between significant other solicitous responses and patient disability for highly satisfied relationships only. In this study, high relationship satisfaction strengthened the association between solicitous responses and patient disability. In addition, relationship satisfaction also moderated the association between solicitous responses and fatigue severity for all patients; the impact of solicitous responses upon fatigue severity increased as relationship satisfaction increased within the dyad. Although these findings may seem counterintuitive upon first inspection, it is possible that the significant other beliefs, emotions, and responses associated with high relationship satisfaction are impacting patient outcomes in a different way compared to those generated in dyads where relationship quality is low.

#### Specific Aspects of Relationship Quality

Goodwin (1997, 2000) identified that in addition to overall relationship adjustment, patient symptom reports were associated with high levels of patient-perceived conflict and reduced significant other empathy. Patient perception of symptom transition, that is, the extent to which the illness or symptoms are perceived to be in a state of change (Goodwin, 2000), was also associated with reduced levels of patient and significant other empathy, reduced significant other support, and increased perceived conflict.

#### Expressed Emotion

Expressed Emotion (EE) is a multicomponent construct that assesses the patient–significant other relationship along various positive and negative dimensions (Vaughn & Leff, 1976). High EE, determined by highly critical or emotionally over-involved (EOI) significant other attitudes, was associated with significantly greater patient fatigue severity at approximately 6 months after entering specialist treatment programs (Band, Barrowclough, & Wearden, 2014). Furthermore, high levels of significant other criticism predicted increased patient depression, and analyses suggested that depression mediated the relationship between high levels of criticism and poorer fatigue outcomes. Examination of significant other subgroups (i.e., parents and partners) suggested that relationship variables are likely to vary when considering the relationship between a parent and child in comparison with examining coupled dyads (Band et al., 2014). This is a potential avenue for future exploration.

## Discussion

This review aimed to evaluate the evidence examining significant other responses to CFS/ME, in addition to the impact that these responses may have upon patient outcomes. Patient affective, cognitive, and behavioral responses have been identified as important in CFS/ME symptom maintenance and perpetuation (Deale et al., 1998; Moss-Morris, 2005), in line with cognitive-behavioral models (Deary et al., 2007; Surawy et al., 1995). The evidence outlined within this review suggests that significant other factors could be incorporated into current cognitive-behavioral models to further develop flexible understanding of CFS/ME perpetuation (Moss-Morris et al., 2013); currently, these theoretical models lack specificity in terms of outlining the interpersonal processes implicated in symptom perpetuation (Deary et al., 2007). Our findings therefore provide an opportunity to develop current understanding of ongoing CFS/ME by highlighting specific ways in which significant other factors impact on symptom perpetuation, and outline clinically important directions for future research within this area.

**Figure 2:**
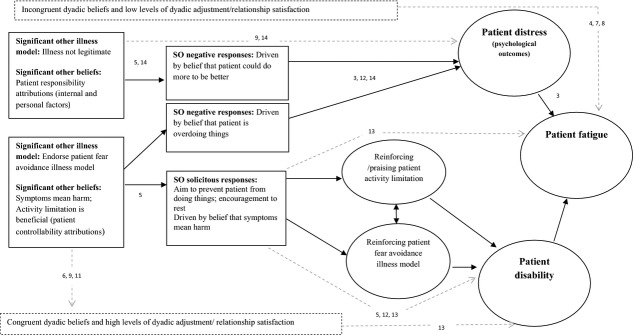
Proposed interpersonal processes involved in symptom maintenance and perpetuation in CFS/ME. *Note*. SO = significant other. Numbers indicate the study source. 1 = Ax (1999); 2 = Ax et al. (2002); 3 = Band et al. (2014); 4 = Blazquez et al. (2012); 5 = Brooks, Daglish, et al. (2013), Brooks, King, et al. (2013); 6 = Butler et al. (2001); 7 = Goodwin (1997); 8 = Goodwin (2000); 9 = Heijmans et al. (1999); 10 = Kelly et al. (1999); 11 = Richards et al. (2006); 12 = Romano et al. (2009); 13 = Schmaling et al. (2000); 14 = White et al. (2006).

We propose that the current evidence highlights the presence of two potentially important interpersonal processes that may usefully extend current theoretical models of CFS/ME, linking significant other beliefs, responses, and aspects of the dyadic relationship with patient illness outcomes (see Figure2). We outline a potential “negative” interpersonal process, characterized by significant other beliefs that attribute responsibility to the patient with respect to illness onset or symptom experience (Brooks, Daglish, et al., 2013; Heijmans et al., 1999; White et al., 2006). We speculate that these significant other beliefs are associated with behavioral responses that have been characterized as negative, unhelpful, or critical within the literature (Band et al., 2014; Romano et al., 2009; White et al., 2006) and propose that poorer patient outcomes are likely to occur as a result of increased patient distress (Band et al., 2014; Romano et al., 2009; White et al., 2006). Significant other beliefs that are incongruent to those held by the patient (Heijmans et al., 1999; White et al., 2006) and poorer relationship quality (Blazquez et al., 2012; Goodwin, 1997, 2000) are further potential mediating factors associated with this interpersonal process.

The findings outlined appear to reflect the wider literature, where the impact of interpersonal relationships has often been guided by attribution theory (Weiner, 1985). Causal attributions are proposed to generate both behavioral and emotional consequences (Weiner, 1985) and have been examined extensively in the context of significant other Expressed Emotion (Barrowclough & Hooley, 2003). Across several diagnostic groups, significant others who make patient responsibility attributions are consistently likely to be rated as critical or hostile (Barrowclough & Hooley, 2003). Further evidence suggests that significant other behaviors are also associated with high levels of criticism, often reflecting attempts to exert control over the patient, symptoms, or undesirable characteristics (Vasconcelose Sa, Wearden, & Barrowclough, 2013). Therefore, the associations described here between significant other beliefs and behavioral responses in CFS/ME are consistent with evidence accumulated across diagnostic groups and theoretical frameworks. In addition, qualitative studies suggest that patients feel more rejected and perceive less support and empathy when the legitimacy of the condition is questioned by their significant others (Dickson et al., 2007). Poorer long-term CFS/ME outcomes have also been documented in association with increased patient depression (Bentall, Powell, Nye, & Edwards, 2002; Wearden, Dunn, Dowrick, & Morriss, 2012).

In contrast, we propose that a second “solicitous” interpersonal process may be important for reinforcing patient fear avoidance illness models (Deale et al., 1998) on both a cognitive and behavioral level. Solicitous-style behavioral responses are related to poorer patient outcomes such as increased levels of disability and fatigue (Brooks, Daglish, et al., 2013; Romano et al., 2009; Schmaling et al., 2000). The impact of solicitous significant other responses may be the result of reduced patient activity, either directly as a result of “encouragement to rest” (Brooks, Daglish, et al., 2013) or indirectly because of a reduction in the number of opportunities patients have to engage in everyday physical activities (Romano et al., 2009; Schmaling et al., 2000). Patient beliefs about the role of activity have been shown to be important for activity limitation (Silver et al., 2002), and the adverse effects of excessive patient resting on symptom maintenance in CFS/ME have been documented within the literature (Deale et al., 1998; Wearden & Emsley, 2013). We propose that significant other responses associated with this interpersonal process may reinforce patient illness models; the limited evidence suggests that significant others and patients tend to hold congruent beliefs in relation to the illness onset and symptom experience (Butler et al., 2001; Heijmans et al., 1999; Richards et al., 2006). Alternatively, solicitous responses may directly reinforce patient activity limitation behavior (McCracken, 2005). In contrast to the “negative” interpersonal process, we suggest that the “solicitous” interpersonal process may be associated with higher relationship satisfaction (Heijmans et al., 1999), increased perceived support, empathy, and lower conflict (Goodwin, 1997, 2000). Finally, however, we speculate that such significant other illness models may also result in critical significant other responses when significant others believe the patient is exacerbating symptoms by doing too much (Band et al., 2014; Vasconcelose Sa et al., 2013). Figure2 depicts a model of the relationships between significant other beliefs, behavioral responses, and the impact on patients as has been discussed above. Not all of the proposed pathways outlined in Figure2 currently have evidence to support them.

The evidence reviewed also offers potential clinical utility, supporting current UK National Institute for Health and Clinical Excellence (NICE) guidelines recommending that significant others should be involved in patient treatment programs where appropriate and also be provided with the information and support they require (NICE, 2007). It has been long recommended that a full exploration of potential perpetuating factors should be made at assessment to identify potential barriers to recovery and that it is particularly important to include family members within the assessment and rehabilitation process (Sharpe, Chalder, Palmer, & Wessely, 1997). The findings presented within this review outline the proposed interpersonal processes that may be beneficial for future significant other–focused interventions to address, such as targeting significant other beliefs and responses that are in contrast to the principles of CBT or GET, as recommended by the UK NICE guidelines (NICE, 2007). Currently, presentation of explanatory models for CFS/ME forms introductory aspects of patient therapeutic programs (Wearden et al., 2010; White et al., 2011); those that are physiologically based may be more acceptable to patient groups (Castell et al., 2011) and may also be beneficial for significant others. The development of intervention components to address unhelpful significant other beliefs (such as those surrounding illness legitimacy or patient symptom focusing and fear avoidance) may inform appropriate significant other responding. This would ensure that well-intentioned responses, such as encouraging patient resting, are in line with current management strategies (Brooks, King, et al., 2013). Previous therapeutic interventions providing family-focused CBT for adolescents with CFS/ME have found it to be helpful in improving patient outcomes over relatively short follow-up periods (Chalder, Deary, Husain, & Walwyn, 2010); family psychoeducation was equally efficacious in improving long-term primary patient outcomes (Lloyd, Chalder, & Rimes, 2012). No such interventions have been published with adults experiencing CFS/ME. However, several efficacious familial interventions have been developed and utilized within other patient groups. Typically, increasing significant other knowledge about the condition, targeting appraisals about the impact of the illness and regarding caring for the patient, in addition to increasing coping resources, have been highlighted as important aspects of these interventions (Addington, McCleery, & Addington, 2005; Pfammatter, Junghan, & Brenner, 2006; Pharoah, Mari, Rathbone, & Wong, 2010).

It has been suggested that the focus of interventions should be on reducing distress and improving well-being for all family members (Lobban et al., 2013). In examining significant other responses to CFS/ME, some of the negative consequences associated with the experience of living with a close relative with CFS/ME have been explored (Ax et al., 2002; Kelly et al., 1999). Aspects of the patient–significant other relationship have been shown to be important for significant other well-being (Brooks, Daglish, et al., 2013), in line with the wider literature suggesting that CFS/ME may impact the whole family (Donalek, 2009). Addressing significant other adjustment may be particularly important, especially since significant other coping strategies have been found to be associated directly with patient coping (Ax, 1999), and considering the evidence linking low dyadic relationship quality and poorer patient outcomes (Blazquez et al., 2012; Goodwin, 1997, 2000). Further research to identify factors that influence coping strategies and identifying effective significant other coping strategies would also be beneficial, particularly early in the illness course, where both patients and significant others experience difficulties understanding and adapting to the illness (Ax et al., 2002; Brooks, King, et al., 2013).

The studies synthesized here have included a variety of significant other samples, including mixed, parent, or partner significant other subgroups. We propose that inclusion of various significant other relationship types is important in representing the diversity of patient experiences of living with CFS/ME. However, careful consideration and comparison of these contextual relationship factors is currently lacking within the current literature; addressing these issues in future empirical studies is warranted in order to advance understanding of CFS/ME perpetuation in the context of significant other relationships.

### Limitations of the Reviewed Literature and Future Recommendations

The findings outlined in this review need to be considered alongside the various methodological issues that have been raised throughout this synthesis. The quality assessment determined there were a number of areas where all studies could have been improved. For example, none of the studies reported consideration of statistical power in calculating the size of samples recruited; these vary between studies despite similar analyses being undertaken.

Several references have been made throughout the synthesis to the properties of the measures used within the literature. Given the wide variety of measurement techniques used, direct comparisons and firm conclusions are difficult to draw from the limited evidence available. Only physical functioning was measured consistently across studies (using the SF-36 subscale). Widely used patient outcome measures, such as those listed in the UK CFS/ME national outcomes database (Collin, Crawley, May, Sterne, & Hollingworth, 2011), may be particularly useful to include in future studies, in addition to CFS/ME-specific significant other measures where possible, such as the FRQ. Other validated measures such as the IPQ or MPI enable associations to be compared with those observed in other patient groups.

An additional limitation is the cross-sectional, largely correlational nature of those studies reviewed. It is recommended that longitudinal relationships between these significant other factors and patient outcomes are explored in future studies. Additionally, alternative methodologies such as experience sampling methodology (e.g., Csikszentmihalyi & Larson, 1987) may be particularly suited to examining the potential fluctuations in symptom experience within CFS/ME. These methodologies offer advantages over traditional self-report techniques, allowing for the assessment of temporal relationships between variables (Palmier-Claus et al., 2011). Additionally, more systematic inclusion of significant other reports of these variables in future research would reduce potential common method variance arising from patient reports of both significant other factors and illness-related outcomes, which must be considered as a potential confound within the current literature.

## Conclusions

This article aimed to critically review studies examining significant other responses to CFS/ME. We found that significant others experience negative consequences following the development of CFS/ME in a close relative; however, these are managed more effectively over time with increasing adjustment to the condition. Many of the studies demonstrated that in general, significant others tend to hold similar beliefs to patients about the condition. Significant other emotional and behavioral responses were also correlated with specific significant other beliefs about the illness. The evidence suggests the presence of two potential interpersonal processes important for poorer patient outcomes, each of which is characterized by differing levels of relationship satisfaction, significant other responses, and associations with patient illness outcomes. We propose that these hypothetical interpersonal processes offer the opportunity to stimulate future research in a systematic, theory-driven approach and may be beneficial in guiding development of intervention components for the benefit of both patients and significant others.
